# Reduction and stabilization of radial neck fractures by intramedullary pinning: a technique not only for children

**DOI:** 10.1186/s40001-016-0210-4

**Published:** 2016-04-12

**Authors:** G. H. Sandmann, M. Crönlein, M. Neumaier, M. Beirer, A. Buchholz, U. Stöckle, P. Biberthaler, S. Siebenlist

**Affiliations:** Department of Trauma Surgery, Klinikum Rechts Der Isar, Technische Universität München, Ismaninger Strasse 22, 81675 Munich, Germany; Department of Orthopaedic Sports Medicine, Klinikum Rechts Der Isar, Technische Universität München, Munich, Germany; BG Unfallklinik Tübingen, Eberhard-Karls- University, Tuebingen, Germany

**Keywords:** Radial neck fractures, Intramedullary pinning, Titanium elastic nail, Radial head fractures

## Abstract

**Background:**

Isolated radial neck fractures occur only in rare cases. The majority of cases are non-displaced or minimally displaced and can be treated conservatively. Conservative treatment, however, might result in secondary displacement and/or malunion. On the other hand, open reduction and internal fixation (ORIF) as standard surgical approach in adults is associated with non-union, implant-related complications and reduced range of motion. For isolated radial neck fractures with an intact radial head, the procedure of centromedullary pinning—as widely used in the treatment of paediatric radial neck fractures—might be an alternative operative technique in adults as well. The purpose of this retrospective case series therefore was to evaluate the functional outcome of radial neck fractures treated by intramedullary pinning.

**Methods:**

Between 02/2009 and 12/2014, a total of eight patients with isolated radial neck fractures (Mason type-III; Judet Type II and III) were treated with centromedullary pinning using titanium elastic nails (TEN). The mean age of the patients was 39 years (range 23–90 years) with a mean interval from injury to surgery of 2.9 days (range 1–7 days). Subjective and objective criteria included patient’s satisfaction, pain rating on a visual analogue scale (VAS) and active range of motion (ROM) compared to the contralateral armside. Functional scoring included the Morrey Elbow Score (MEPS), the QuickDASH and the Elbow Self Assessment Score (ESAS). Furthermore, follow-up radiographs were evaluated.

**Results:**

Seven of the eight patients were available for follow-up after a mean of 36 months (range 6–64 months). Patients’ satisfaction was rated very good in four cases, good in two cases and sufficient in one case. An unrestricted active ROM compared to the contralateral side for extension-flexion arc and for pronation-supination-arc with full strength was rated in all cases. The Elbow Self Assessment Score was 98.52 ± 1.95 (range 96–100), the calculated Mayo elbow performance score was 95.71 ± 7.32 (range 85–100) and the QuickDASH score was 6.81 ± 10.42 (range 0–27). There were no complications as infection, non-union, heterotopic ossifications or secondary loss of reduction of the radial head. Only one patient complained about pain resulting from an affection of the superficial radial nerve.

**Conclusion:**

In the present cohort, good to excellent results without relevant complications were seen. The technique of intramedullary pinning as described in the treatment of isolated radial neck fractures in children represents a suitable and reliable method in adults as well. In selected cases, this technique can be recommended as an alternative, minimal-invasive approach to the radial head plate osteosynthesis.

## Background

In adults, isolated radial neck fractures are very rare with an incidence of 1 % of all fractures [1]. There is no separate classification of these fracture types and the therapeutic management is controversial depending on fracture displacement. The majority of slightly displaced radial neck fractures are treated conservatively with early initiation of physical therapy [2]. However, displaced fractures in adults have to be managed surgically as the correction potential is limited compared to that in children. For severely displaced fractures, open reduction and internal fixation (ORIF) by locked plating is recommended as the treatment of choice nowadays [3]. Nevertheless, ORIF with the potential harm of the surgical approach due to an affection of the vascularization of the radial head and implant-related problems potentially affecting the forearm rotation seems not to be adequate in slightly displaced fractures [4, 5].

In paediatric surgery—based on the classification of Judet—displaced radial neck fractures with an angulation of more than 30° are treated surgically [6–8]. In most cases, the procedure of intramedullary pinning is used. This minimal-invasive technique with a limited risk of the surgical approach leaves the proximal radio-ulnar joint intact, allows a reliable reduction of the radial head, and is proven with good to excellent results in children and adolescents [9–12].

Besides reported outcomes of standard open procedures (ORIF), there is only a single case published in the literature evaluating the results of intramedullary pinning of radial neck fractures in adults [13]. Therefore, the aim of this retrospective case series was to determine the functional results of this technique performed in mature elbow surgery.

## Methods

### Patients

Between February 2009 and December 2014, a total of eight patients were treated with intramedullary pinning using titanium elastic nails (TEN). All patients had suffered from an isolated radial neck fracture without clinical signs of joint instability (type-III-fractures according to Mason’s classification [6]). Three of eight treated fractures were initially notable displaced due to their injury pattern. The remaining five fractures were slightly displaced after injury but rated as unstable with a high risk of secondary displacement (due to metaphyseal comminution). With a mean of 36 months (range 6–64 months; minimum 6 months) seven patients (two men, five women) returned for follow-up survey. One patient was not available due to bad general conditions.

### Surgical technique

**Fig. 1:**
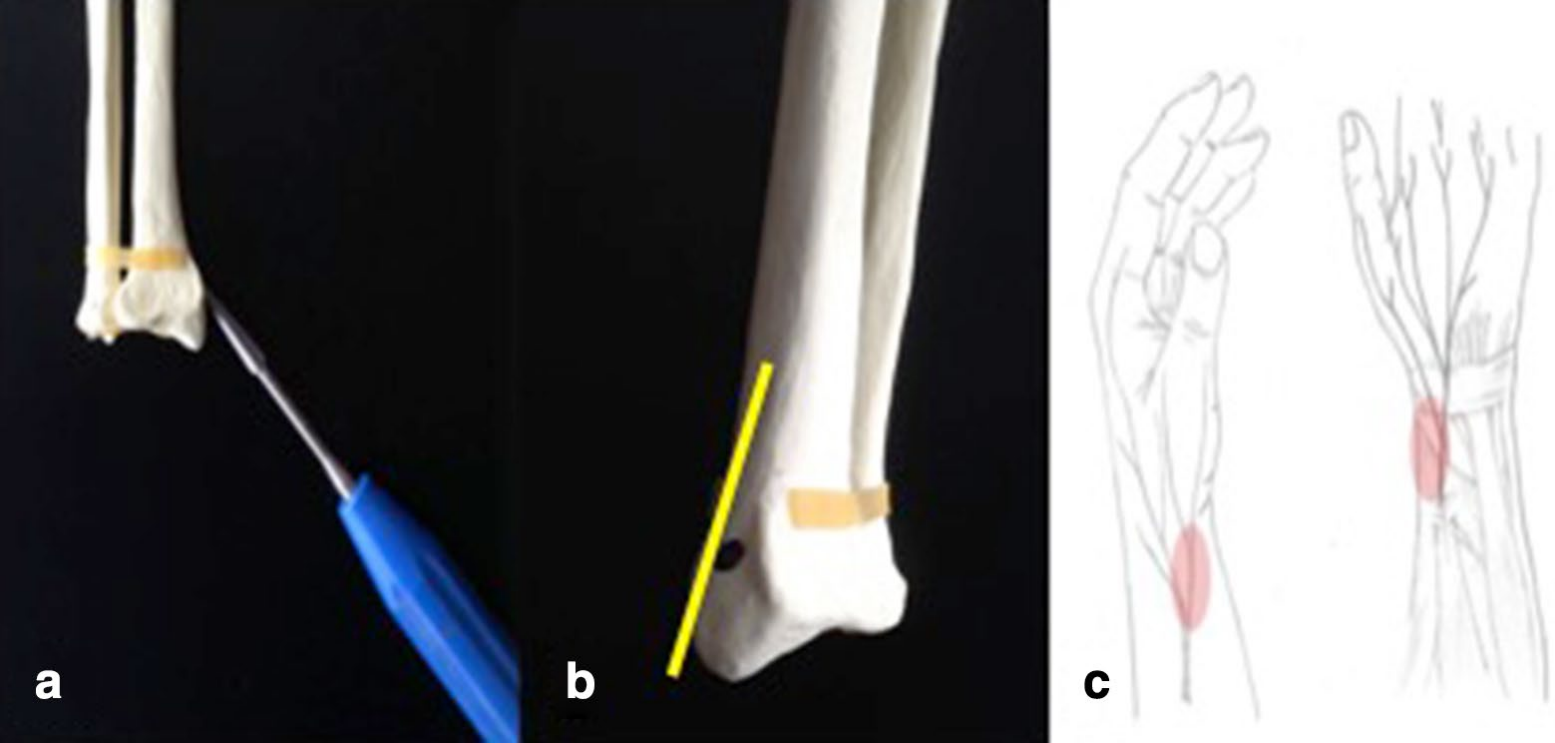
illustration of the correct entry point of the TEN. The correct entry point of the TEN on a sawbone (**a**) is illustrated in this figure. The cortex has to be exposed and perforated using a Pfriem-type trocar (**a**/**b**), by taking care not to injure the superficial branch of the radial nerve (**c**)

The operative treatment of intramedullary pinning was performed using the technique described by Metaizeau [6]. In all cases, the patient was placed supine with the injured arm on a radiolucent table under general anaesthesia. Before skin incision, fluoroscopy was performed to confirm joint stability. As entry point for the TEN, the metaphyseal zone of the styloid process of the distal radius was marked under image intensifier (see Fig. 1). After a 0.5-cm skin incision and soft tissue dissection, the superficial branch of the radial nerve was visualized and carefully retracted. The lateral radial cortex was exposed and perforated using a drill with a diameter of 2.5 mm. Thus, the TEN was introduced into the intramedullary canal and following the displaced radial head was reduced with gently rotational movements under fluoroscopic control. According to the width of the intramedullary canal, one or two TEN (Fa. DepuySynthes, Umkirch, Germany) with different thicknesses were used for closed intramedullary pinning in all patients (Table 1). All operations were performed by experienced upper extremity surgeons with a mean interval from injury to surgery of 2.9 days (range 1–7 days). No additional surgical procedures were necessary in any case.

**Table 1:** patients’ collective

Case	Sex (age/years)	Injury pattern	Interval trauma - surgery (days)	Used implants	Follow-up (months)	Patient’s satisfaction	ESAS	VAS	MEPS	*Quick*DASH	ROM	Bony healing	Complications
1	Male (89)	Direct impact on the forearm	1	1 × 3.0 mm TEN	Lost to follow-up								
2	Male (33)	Fall from a standing height	3	2 × 2.0 mm TEN (DepuySynthes)	64	1	100	0	100	0	Equal to contralateral elbow	Yes	None
3	Female (47)	Fall from a wall of less than 3 m height	2	1 × 2.0 mm TEN (DepuySynthes)	63	4	96	4	85	27	Equal to contralateral elbow	Yes	Neurapraxia of the cutaneus branch of the radial nerve
4	Male (50)	Fall from a ladder of less than 3 m height	1	2 × 2.0 mm TEN (DepuySynthes)	54	1	100	0	100	0	Equal to contralateral elbow	Yes	None
5	Female (43)	Fall from a standing height	5	2 × 2.0 mm TEN (Synthes	43	1	100	0	100	0	Equal to contralateral elbow	Yes	None
6	Female (25)	Fall from a scooter on the outstretched arm	2	1 × 2.0 mm TEN (DepuySynthes)	12	2	98	0	100	7	Equal to contralateral elbow	Yes	None
7	Female (26)	Boulder injury, fall from less than 3 m on the outstreched arm	2	1 × 2.5 mm TEN (DepuySynthes)	7	1	100	0	100	0	Equal to contralateral elbow	Yes	None
8	Female (26)	Boulder injury, fall from 3 m on the outstreched arm	7	1 × 2.5 mm TEN (DepuySynthes)	6	2	96	0	85	14	Equal to contralateral elbow	Yes	None

The patients’ collective is shown exemplarily. Outcome is rated by subjective and objective criteria including patient’s satisfaction, pain rating on a VAS and active ROM. Functional scoring included MEPS, QuickDASH and ESAS. Furthermore, follow-up radiographs were evaluated

For postoperative management, all patients were immobilized with a plaster cast for 2 days. Active-assisted range of motion was allowed immediately after surgery under physiotherapeutical control. Sporting activities were restricted for 3 months.

### Evaluation

Personal interviews and elbow scoring were carried out by an independent investigator (MC) not involved in the initial surgical management. All patients gave written informed consent prior to being included to the study. The study protocol was approved by the institutional ethics committee (IRB 314/15).

For subjective evaluation, patients rated their satisfaction for elbow use on a scale of 1–6 (1—very good; 2—good; 3—satisfied; 4—sufficient; 5—insufficient; 6—poor) patients additionally fulfilled the Elbow Self Assessment Score (ESAS) [14]. Objective assessment consisted of a physical examination for active elbow range of motion (ROM) for extension and flexion as well as forearm rotation on the injured and uninjured arm. Moreover, sensomotoric disturbances and postoperative complications were recorded. Functional scoring included a visual analogue scale (VAS) for pain rating, the Mayo Elbow Performance Score (MEPS) [15] and the shortened disabilities of the arm, shoulder and hand questionnaire (*Quick*DASH) [16, 17]. Postoperative x-rays were evaluated with special respect to bony healing, heterotopic ossifications and the alignment of the radiocapitellar joint as well. All results were presented as mean (Ø) ± standard deviation values.

## Results

All results are summarized in Table 1. At time of follow-up, patients rated their satisfaction for elbow function as very good in four cases and good in two cases. Only one of the patients rated the postoperative result as sufficient.

The active ROM was unrestricted in all patients when compared to the unaffected side. Similarly, the strength for flexion–extension and pronation–supination was identical in seven of seven evaluated patients. None of the patients was affected in daily life or sporting activities. In the present cohort, there was only one minor complication (Table 1, case 3). This patient complained about an ongoing affection of the superficial radial nerve at the area of the entry point of the TEN. Pain has been described in this case with an intensity of four on the VAS during follow-up examination.

For functional scoring, the VAS for pain was Ø 0.6 (range 0–4), the MEPS was Ø 95.71 ± 7.32 (range 85–100) representing two good and five excellent results and the *Quick*Dash revealed good to excellent results with Ø 6.81 ± 10.42 points (range 0–27). Consequently, the self-evaluation-score (ESAS) was Ø 98.52 ± 1.95 (range 96–100) indicating a non-restricted elbow function.

**Fig. 2:**
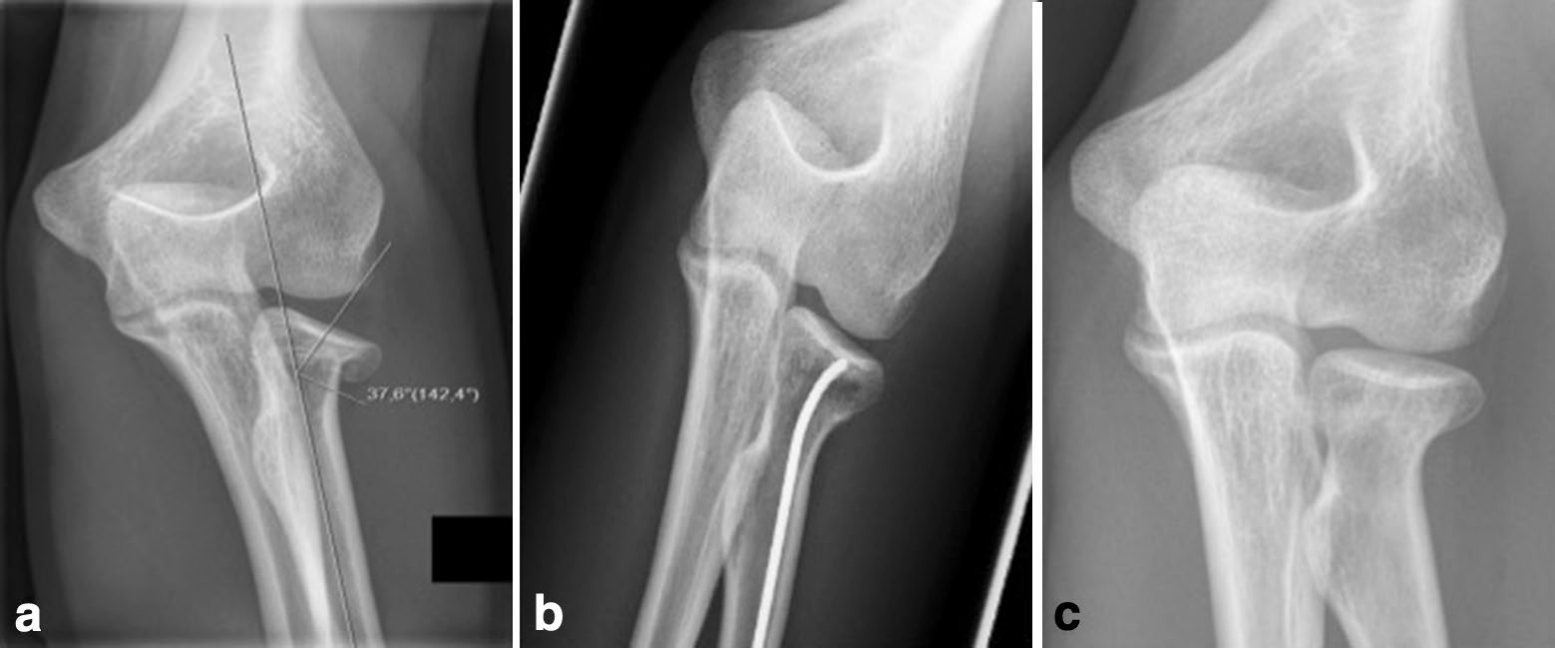
a.p. X-rays of patient nr 7 (see Table 1). This figure shows the a.p. X-rays of patient number 7. The patient suffered from a displaced Mason type III radial neck fracture (**a**). The postoperative results (**b**) following intramedullary pinning show a very good alignment. After implant removal (**c**) bony healing could be obtained, without a loss of alignment

At time of follow-up evaluation, in all cases the TEN had already been removed with osseous healing of the radial neck. Heterotopic ossifications or avascular radial head necrosis were not seen in any patient. Follow-up radiographs showed a congruent radiocapitellar joint in all cases without any secondary loss of reduction or secondary displacement of the radial head (Fig. 2).

## Discussion

Several studies have shown that the procedure of closed reduction and intramedullary pinning of isolated radial neck fractures is a safe technique leading to reliable results in children.

The present good to excellent functional results without affecting the elbow range of motion or patients’ activities of daily life support the statement that this approach can also be an alternative surgical procedure in the treatment of displaced radial neck fractures in adults. The advantages of this minimally-invasive technique are found in the avoidance of implant—related complications like screw dislocation or perforation affecting the proximal radio-ulnar joint. In addition, the ORIF is associated with a higher rate of avascular necrosis [18, 19], proximal synostosis [20], heterotopic ossifications [21], infection and loss of ROM [22]—especially in those cases where the plate has to be positioned out of the safe zone due to fracture pattern [4].

However, the indications for the described technique differ from those of open approaches. We see the indication for the closed reduction and intramedullary pinning especially in those cases where the ORIF procedure might be an overtreatment and the conservative treatment might just not be enough as the dislocation of the radial head can lead to an incongruity of the proximal radio-ulnar joint resulting in limited forearm rotation and/or leading to early onset osteoarthritis. Based on present data, the described technique represents a reliable option for type II and III fractures according to Judet [8] in adults. However, owing to the experiences of this case series, it has to be clearly stated that, in mature patients, the intramedullary procedure should be performed within the first week after trauma to obtain anatomical reduction of the displaced radial head. Failing this, the closed reduction by the TEN may not be possible due to the initiating fracture healing.

Nevertheless, there are reports in paediatric traumatology showing that even severely displaced fractures type III to IV according to Judet classification can be treated by use of this technique, but whether this works in adults as well has not been shown yet [23–25]. In those cases, the use of an additional percutaneous K-wire as a tool for reduction of the radial neck might be necessary [26].

Still this technique is not without potential complications and especially care has to be taken to avoid an affection of the superficial radial nerve as we found one patient complaining of hyperaesthesia of the dorsal thumb and the index finger. In addition, a second surgery is necessary for the TEN removal.

Till now we used this technique only in radial neck fractures with intact radial head as we are confident that ORIF of radial head/radial neck fractures with screws and plates is the treatment of choice. However, a sort of hybrid technique seems conceivable: after open reduction of the radial head with screws in partial radial head fractures, the radial neck component is stabilized by use of an intramedullary pin. This technique might help reduce the problems caused by the radial head plates affecting range of motion, especially in those cases, where the plate has to be used out of the safe zone. In addition, one would notice ligamentous lesions or postero-lateral instabilities caused by initial fall on the outstretched arm [27]. Therefore, especially lesions of the MCL have to be excluded prior the presented surgical procedure due to the important role of the MCL as a primary joint stabilizer in cases of radial head insufficiency. Future studies will show, whether the combination of a limited open reduction with intramedullary pinning is possible and helps reduce complications.

Though this is the first study to show that the transfer from paediatric traumatology to adults is possible, there are still some limitations. As isolated fractures of the radial neck not affecting the head are rare and therefore the number of patients who could be treated by this technique is limited, the number of patients presented in this study is small. Moreover, it has to be clearly stated that the rotation of the radial head is difficult to control during this minimal-invasive procedure, especially if only one single TEN is used. To address rotational malalignments, it could be possible to fix or to manipulate the radial head percutaneously from the lateral side.

Nevertheless, this study has several strengths. Except one, all patients were available with a mean of 36 months of follow-up and a complete assessment of objective functional parameters and elbow scoring. Moreover, this is the first case series evaluating this surgical technique applied in adults. Although we found good to excellent results for the presented technique, a randomized controlled trial with long-term follow-up may provide further guidelines for the optimal management of these fracture types. Thinking of the rare incidence of radial neck fractures, this kind of study will only be possible in a multi-center setup.

## Conclusion

The current study shows that the treatment of isolated radial neck fractures using an intramedullary pin is a reliable and safe surgical technique in adults leading to good to excellent clinical results. Compared to ORIF, this surgical technique has a minor complication rate, though crucial care has to be taken to avoid any harm to the superficial radial nerve during the approach. Future research is needed to find out the best treatment option for this rare kind of elbow injuries.

## Abbreviations

ORIF: open reduction and internal fixation
TEN: titanium elastic nail
VAS: visual analogue scale
ROM: range of motion
MEPS: Morrey Elbow Performance Score
QuickDASH: shortened disabilities of the arm, shoulder and hand questionnaire
ESAS: Elbow Self Assessment Score

## References

[1] Duckworth AD, Clement ND, Jenkins PJ, Aitken SA, Court-Brown CM, McQueen MM (2012). The epidemiology of radial head and neck fractures. J Hand Surg Am.

[2] Schmidt-Horlohe K, Siebenlist S, Stockle U, Pichl J, Hoffmann R (2011). Fractures of the radial head and neck. Z Orthop Unfall.

[3] Burkhart KJ, Gruszka D, Frohn S, Wegmann K, Rommens PM, Eicker CM, Muller LP (2015). Locking plate osteosynthesis of the radial head fractures: clinical and radiological results. Unfallchirurg.

[4] Ries C, Muller M, Wegmann K, Pfau DB, Muller LP, Burkhart KJ (2015). Is an extension of the safe zone possible without jeopardizing the proximal radioulnar joint when performing a radial head plate osteosynthesis?. J Shoulder Elbow Surg.

[5] Kang HJ, Shin SJ, Kang SS (2012). Nonunion of the radial neck following operative treatment for displaced radial head and neck fractures. Acta Orthop Belg.

[6] Metaizeau JP, Schmtt M (1980). Réduction et fixation des fractures et decollement épiphysairesde la tete de radial par broche centromedullaire. Rev Chir Orthop.

[7] Prathapkumar KR, Garg NK, Bruce CE (2006). Elastic stable intramedullary nail fixation for severely displaced fractures of the neck of the radius in children. J Bone Joint Surg Br.

[8] Judet H, Judet J (1974). Fractures and orthopedicque de l´enfant.

[9] D’Souza S, Vaishya R, Klenerman L (1993). Management of radial neck fractures in children: a retrospective analysis of one hundred patients. J Pediatr Orthop.

[10] Metaizeau JP, Lascombes P, Lemelle JL, Finlayson D, Prevot J (1993). Reduction and fixation of displaced radial neck fractures by closed intramedullary pinning. J Pediatr Orthop.

[11] Ugutmen E, Ozkan K, Ozkan FU, Eceviz E, Altintas F, Unay K (2010). Reduction and fixation of radius neck fractures in children with intramedullary pin. J Pediatr Orthop B.

[12] Eberl R, Singer G, Fruhmann J, Saxena A, Hoellwarth ME (2010). Intramedullary nailing for the treatment of dislocated pediatric radial neck fractures. Eur J Pediatr Surg.

[13] Serbest S, Gurger M, Tosun HB, Karakurt L (2015). Closed reduction and intramedullary pinning in the treatment of adult radial neck fractures: a case report. Pan Afr Med J.

[14] Beirer M, Friese F, Lenich A, Crönlein M, Sandmann GH, Biberthaler P, Kirchhoff C, Siebenlist S (2015). The Elbow Self-Assessment Score (ESAS): development and validation of a new patient-reported outcome measurement tool for elbow disorders. Knee Surg Sports Traumatol Arthrosc.

[15] Morrey BF, An KN, Chao EY, Morrey BF (1985). Functional evaluation of the elbow and its disorders. the elbow and its disorders.

[16] Germann G, Harth A, Wind G, Demir E (2003). Standardisation and validation of the German version 2.0 of the disability of arm, shoulder, hand (dash) questionnaire. Unfallchirurg.

[17] Gummesson C, Ward MM, Atroshi I (2006). The shortened disabilities of the arm, shoulder and hand questionnaire (QuickDASH): validity and reliability based on responses within the full-length DASH. BMC Musculoskelet Disord.

[18] Young S, Letts M, Jarvis J (2000). A vascular necrosis of the radial head in children. J Pediatr Orthop.

[19] Girard JY, Rogez JM, Robert R, Leborgne J (1995). Vascularisation of the head of the radius in the adult. Surg Radiol Anat.

[20] Jupiter JB, Ring D (1998). Operative treatment of post-traumatic proximal radioulnar synostosis. J Bone Joint Surg Am.

[21] Foruria AM, Augustin S, Morrey BF, Sanchez-Sotelo J (2013). Heterotopic ossification after surgery for fractures and fracture-dislocations involving the proximal aspect of the radius or ulna. J Bone Joint Surg Am.

[22] Cikes A, Jolles BM, Farron A (2006). Open elbow arthrolysis for posttraumatic elbow stiffness. J Orthop Trauma.

[23] Al-Aubaidi Z, Pedersen NW, Nielsen KD (2012). Radial neck fractures in children treated with the centromedullary Metaizeau technique. Injury.

[24] Nawabi DH, Kang N, Amin A, Curry S (2006). Centromedullary pinning of radial neck fractures: length matters!. J Pediatr Orthop.

[25] Zimmerman RM, Kalish LA, Hresko MT, Waters PM, Bae DS (2013). Surgical management of pediatric radial neck fractures. J Bone Joint Surg Am.

[26] Cha SM, Shin HD, Kim KC, Han SC (2012). Percutaneous reduction and leverage fixation using K-wires in paediatric angulated radial neck fractures. Int Orthop.

[27] Anakwenze OA, Kancherla VK, Iyengar J, Ahmad CS, Levine WN (2014). Posterolateral rotatory instability of the elbow. Am J Sports Med.

